# Identifying technical aliases in SELDI mass spectra of complex mixtures of proteins

**DOI:** 10.1186/1756-0500-6-358

**Published:** 2013-09-08

**Authors:** John C Whitin, Srinivasa Rangan, Harvey J Cohen

**Affiliations:** 1 Department of Pediatrics, Stanford University School of Medicine, 300 Pasteur Drive, Stanford, CA USA; 2 Department of Electrical Engineering, Stanford University, Stanford, CA USA

## Abstract

**Background:**

Biomarker discovery datasets created using mass spectrum protein profiling of complex mixtures of proteins contain many peaks that represent the same protein with different charge states. Correlated variables such as these can confound the statistical analyses of proteomic data. Previously we developed an algorithm that clustered mass spectrum peaks that were biologically or technically correlated. Here we demonstrate an algorithm that clusters correlated technical aliases only.

**Results:**

In this paper, we propose a preprocessing algorithm that can be used for grouping technical aliases in mass spectrometry protein profiling data. The stringency of the variance allowed for clustering is customizable, thereby affecting the number of peaks that are clustered. Subsequent analysis of the clusters, instead of individual peaks, helps reduce difficulties associated with technically-correlated data, and can aid more efficient biomarker identification.

**Conclusions:**

This software can be used to pre-process and thereby decrease the complexity of protein profiling proteomics data, thus simplifying the subsequent analysis of biomarkers by decreasing the number of tests. The software is also a practical tool for identifying which features to investigate further by purification, identification and confirmation.

## Background

Investigations in genomics and proteomics deal with large datasets, and statistical methods are being developed to decrease the complexity of the datasets. Examples of these investigations include protein profiling by mass spectrometry in biomarker discovery studies, in which complex samples are often fractionated prior to analysis. A commonly used method of analysis is to control the fraction of false-positives among significant results (false discovery rate, FDR) [1,2]. While it is important to discover whether biomarkers correlate biologically with each other, strongly correlated peaks or features (due to multiple fractions being examined or other technical issues) usually lead to uncertainty in the estimate of FDR [3], and do not add to finding new biomarkers. Thus, it would be useful to deal with correlations in the analyses of protein profiling mass spectra, as obtained using surface enhanced laser desorption ionization-time of flight mass spectrometry (SELDI-TOP MS).

Biomarker discovery studies using SELDI-TOF-MS will usually consist of many spectra - different samples, often with spectra of each sample using multiple analysis parameters (instrument parameters optimized for proteins of different sizes), and sometimes with spectra of chromatographically fractionated pre-processing of samples to decrease the complexity of the samples.

Protein profiling studies often produce features that strongly correlate. Groups of peaks (features) may have similar, but not identical m/z values, appearing in spectra acquired at different laser energies, from different chromatographic fractions of samples, or even at mass multiples that might indicate different ionizations or protein aggregates. In addition there could be biological correlations such as proteins without and with post-translational modifications [4-6]. We have previously developed a clustering algorithm for dealing with correlations in protein profiling SELDI-TOF proteomic data, such as those found in SELDI biomarker discovery studies [7]. Our previous clustering technique was based on representing each feature (mass spectrum peak) as a vector, with each element of the vector representing a measurement of a sample. The technique creates mean-centered unit vector centroids, and uses measurement noise (replicate value variance, not instrument noise) to determine the feature weights when calculating centroids and the optimal number of clusters at a given variance. However, that clustering technique does not draw a distinction between peaks that biologically correlate and peaks that are “technical aliases” of a single feature. Using many elements of our clustering software, we have developed an algorithm that that has been modified to identify and cluster the “technical aliases” in protein profiling datasets. The clusters are then represented by centroids that are calculated by taking a noise-weighted average of the individual features [7]. Downstream statistical analysis, such as multi-hypothesis testing, can then be applied to the clustered dataset directly, eliminating multiple analyses of the same protein. The aim of technical alias clustering is to decrease the subjectivity of identifying peaks that represent proteins with different charges and aggregates of proteins. A rational way to group technically correlated features in a biomarker dataset will identify peaks representing the same protein in different spectra (whether from different laser energies, chromatographic fractions or peaks of the same protein with different ionizations) decrease the number of statistical tests and aid biological interpretation of the data.

## Results and discussion

**Figure 1:**
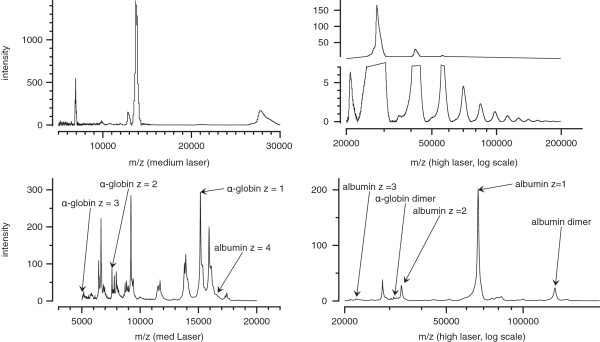
**Examples of mass spectra of purified transthyreting and complex mixtures of proteins.** In the upper spectra, purified transthyretin was applied to a CM10 ProteinChip array and spectra at medium (left) and high (right) laser energies were acquired. Peaks for transthyretin with z = 1 and 2, and transthyretin dimers are prominent in the medium laser spectrum, while aggregates of 2–9 transthyretin molecules can be seen at high laser energy. The lower spectra are averaged from 90 individual spectra (45 samples in duplicate) of the pH4 extract from Q anion exchange chromatographed plasma samples applied to CM10 ProteinChip arrays. Peaks for α-globin and albumin that were used to develop mass windows for the algorithm are annotated.

SELDI-TOF mass spectra of a purified protein demonstrate the presence of peaks representing the protein with single and multiple charges, as well as aggregates of the protein. As an example, peaks representing human transthyretin with one, two, and three positive charges are present in SELDI mass spectra of the purified protein, with peaks attributable to aggregates of up to nine transthyretin molecules also identified (Figure 1). Mass spectra of complex mixtures of proteins have numerous peaks, making the identification of the protein peaks with z > 1 and peaks representing protein aggregates more challenging. In a single spectrum, most experienced researchers can easily identify the parent protein peak with z = 1, and will recognize other peaks as technical aliases (z = 2 or 3) or aggregates of the parent protein peak. The SELDI mass spectrometer vendor (Bio-Rad) provides a software feature to identity likely aliases in a given spectrum, although the algorithm is not disclosed. The widely used and useful SELDI-TOF spectrum processing and peak finding software PROcess can also identify technical aliases in a given spectrum (an R package available in the Bioconductor suite) [8]. In contrast to Bio-Rad’s software, the PROcess software provides a mass window parameter that can be modified by the user.

### Algorithm

**Figure 2:**
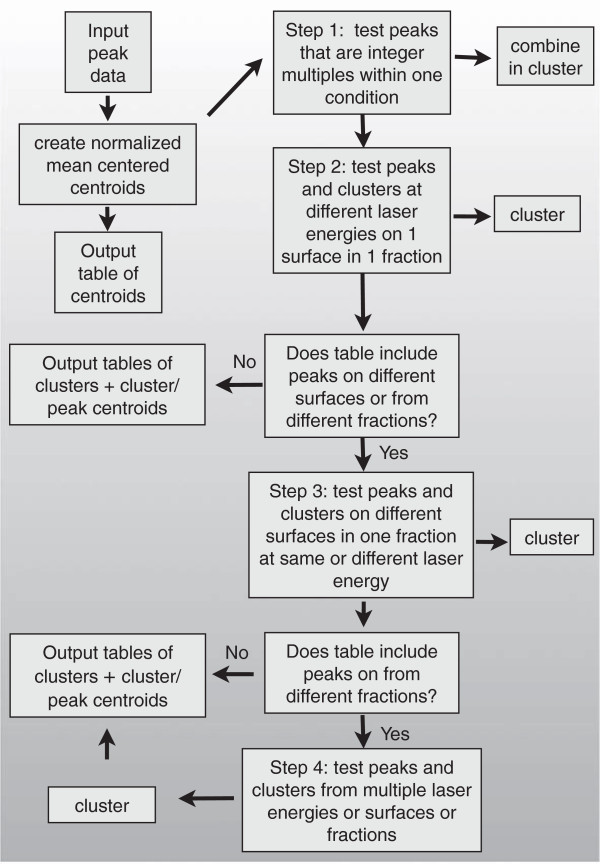
Diagram of the algorithm for technical clustering.

The software is written in R. The goal of the algorithm is to find sets of clusters that group protein peaks that represent the same protein presenting in spectra of several samples as multiple peaks, i.e. technical aliases. Protein profiling biomarker discovery studies might include spectra from different laser energies, chromatographic fractions, or SELDI ProteinChip array surfaces. The clustering of peaks is an iterative process consisting of four steps (Figure 2).

Before the first step, the table of peak intensities is converted to a table of normalized mean centered unit centroids, along with the variance (noise) of the replicate measurement of each sample. Missing values in the data are imputed in the software using the k-nearest neighbor method in PAM software(R package pamr) [9,10]. This table of normalized centroids without clustering is written as an output file that can be used in a downstream statistical test such as FDR analysis. The algorithm then clusters the normalized peak centroids.

### Correlation test

The algorithm represents each peak as a feature vector with each element of the vector representing a measurement on a particular sample.

For peaks that are technical aliases of each other, we expect to find correlation between peak intensities of the individual samples studied. For example, if the easily recognizable albumin peak of approximate mass 66,000 is very high in one sample (relative to the other samples), one might expect that the albumin peak with z = 2 (33,000) would also be very high in that sample (relative to the other samples) (Figure 1). To test this correlation we use the following procedure:

Peaks that are candidates for being technical aliases of each other are identified (see below). We obtain the ratio of the intensities of both peaks for corresponding samples. Thus, we obtain a ratio value for each sample in our dataset.

We then compute the coefficient of variation (CV) of these values. The CV serves as a measure of the dispersion of the data. We set a threshold on the CV value, and if the CV value is less than this threshold, the two peaks are classified as being technical aliases of each other and constitute a cluster. The variance threshold for the clustering is a parameter that can be set in the algorithm.

### Selecting peaks for evaluation as aliases

In step 1, aliases from spectra within one laser energy, on one surface, for one fraction are clustered. Each peak in this set is compared with other peaks in the same set to see if the ratio of the peak locations is an integer multiple. Since the mass values are unlikely be exact multiples of each other, we define an interval within which the mass ratios should lie if the peaks are technical aliases. For example, if we were considering a peak that was located at an apparent mass of 66,000, then a peak of approximately 33,000 would be a possible candidate for a technical alias with z = 2. For each peak, we search in a window centered at that peak to check if there are peaks at a similar location but in a different fraction, array, or laser energy set. The size of this window is a percentage of the peak location itself and is specified in Table 1 for the mass range of 2,000 – 200,000. As shown in the table, the size of the window increases with increasing apparent mass of the peak. For the example of a primary peak of 66,000, the window for a possible technical alias in which z = 2 would be 32,918 – 33,082 (a window of 0.5% of mass). These values were empirically developed to be appropriate for the Bio-Rad PCS4000 mass spectrometer used for SELDI biomarker discovery studies, and are influenced by the mass calibration, mass accuracy and resolution of the instrument. These values were developed using peak intensities derived from SELDI-TOF spectra from a study consisting of 1537 peaks derived from 45 samples. Each sample was processed in duplicate, yielding five chromatographic fractions (Q anion exchange chromatography), applied to two SELDI proteinchip array surfaces (CM10 and H50), and spectra acquired at three laser energies optimized for small, medium, and large proteins (2,700 spectra). The sizes of the mass windows were empirically developed by optimizing the clustering of selected prominent and previously identified proteins (Table 1). As a demonstration, two spectra for medium- and high-laser energy of Fx4 Q extract (pH 4) on CM10 ProtinChip arrays are shown in Figure 1 (spectra are averaged spectra for 90 individual spectra using our Simultaneous Spectrum Analysis software [11]). The peak locations for albumin and α-globin are annotated, for z = 1, 2, and 3, and protein dimers.

**Table 1:** Size of search window as a function of the peak location

**Peak m/z**	**Size of window**	**Proteins used**
	**(as % of mass)**	**(mass with z = 1)**
2000 < m/z < 10,000	0.1%	Apo CI (6,630); Apo CI (−2) (6,432);
10,000 < m/z < 30,000	0.15%	α-globin (15,126); β-globin (15,867)
30,000 < m/z < 70,000	0.5%	Albumin (66,471); transferrin (75,101)
100,000 < m/z	1.0%	Albumin dimers; transferrin dimers

As can be seen in Figure 1, peaks of prominent proteins with z > 3 are difficult to identify in SELDI spectra of complex protein mixtures that are common in biomarker discovery studies and aren’t considered. Once we find candidate peaks that are aliases, we check the variance between the samples of both peaks before concluding that they are actual technical aliases.

In step 2, the technical aliases in spectra obtained at different laser energies on one surface in one fraction are determined by the same clustering test. For example, if the peaks of similar size in spectra obtained in separate spectra obtained using different laser and acquisition parameters (e.g. optimized for small- and medium-sized proteins), the same variance test of peak correlation is performed. If the study uses unfractionated samples on a single surface, the analysis finishes after these first two steps and creates the output files.

If the input table contains peaks from spectra obtained on different surfaces (e.g. CM10 and H50 ProteinChip arrays), the algorithm continues to step 3. In this step technical aliases in spectra from surfaces with different chemistries at the same or different laser energies within a single chromatographic fraction are evaluated. For the given variance threshold, the peaks may be clustered or added to clusters from previous steps.

If the input table contains peaks from spectra obtained from different chromatographic fractions, the algorithm continues to step 4. In this step peak centroids may be clustered with peak centroids from multiple laser energies or multiple surfaces in the same or multiple chromatographic fractions, or added to existing clusters.

### Cluster centroids

Once the peaks that are technical aliases of each other are identified, each cluster is represented by a “centroid” that best captures the features in that cluster. The centroid is computed by taking the noise-weighted average of individual features in the cluster and is a mean-centered unit vector as described in [7]. The software returns two results files:

1) A list of the clusters and the peaks that constitute the clusters is written. For each cluster in the table of clusters, the weighted (by measurement noise) contribution of the components of the cluster and the average variance of each cluster component is reported. While the clusters of technical aliases are developed without regard to any groupings of the samples, this table also returns the variance of the components of the cluster for each group of samples in the dataset, solely for information purposes.

2) A table of centroids for the peaks and clusters for each sample is written. These centroids can then directly be used in downstream statistical analyses.

### Implementation: unfractionated samples

We demonstrate the algorithm using data from two biomarker discovery studies. The first dataset comes from a study of rat cerebrospinal fluid (CSF) in an experimental primary glioma model [12]. The spectra were processed and the table of peaks was developed as described for algorithm steps 1 and 2, because the samples were not chromatographically fractionated, and were applied to only one SELDI surface (CM10). Spectra were obtained at three laser energies optimized for mass ranges of 2,000 – 10,000 (low), 5,000 – 30,000 (medium), and 20,000 – 200,000 (high).

The input table of peaks extracted from the spectra of 45 samples consisted of 247 peaks, with duplicate values for each sample. The first four columns of the input table include the fraction name (Fx0 in this case), laser energy (low, med, or high), SELDI ProteinChip array type (CM10 in this case), and a text string of the mass of the peaks (formatted as exported by the vendor’s software ProteinChip Data Manager, v 3.51). The algorithm uses the first three columns for grouping the features for the different steps of the clustering, and converts the text string of the mass to a numeric mass.

**Figure 3:**
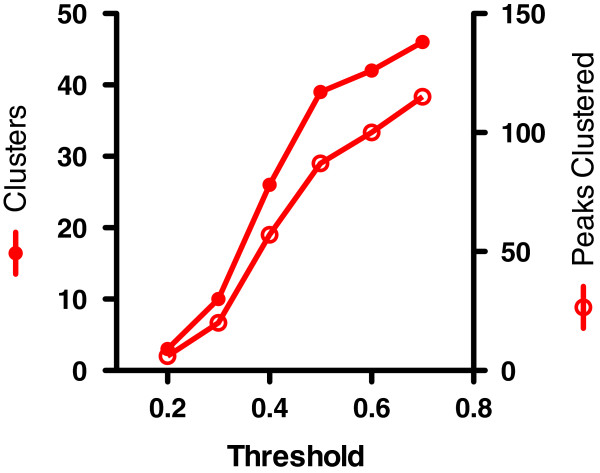
**Number of technical clusters and clustered peaks from unfractionated CSF dataset.** The Technical Clustering software was applied to a dataset of 247 peaks from 45 unfractionated CSF samples in a biomarker discovery dataset at the indicated variance threshold. The number of technical clusters (solid red circles) and number of peaks clustered (open red circles) are plotted.

These data were clustered for technical aliases with this algorithm using variance thresholds ranging from 0.2 – 0.7, and the results are shown in Figure 3. Intuitively one expects that few clusters will be created when the tolerance for noise in the data is low (low variance threshold). No peaks are clustered at thresholds < 0.2, and the number of clusters and peaks within clusters increases as the stringency of variance is decreased (variance threshold is increased).

At a variance threshold of 0.5, 87 of 247 peaks were grouped into 39 clusters, yielding 199 features (cluster centroids plus the peak centroids not clustered at this threshold). Since this was a study of unfractionated CSF with spectra acquired for samples on only one SELDI surface, the results come from only steps 1 and 2 of the algorithm. These centroids were then processed with a downstream statistical test, the Mann–Whitney *U*-test coupled with false discovery rate (FDR) analysis [13]. Before technical clustering, 61 of 247 peaks (24.7% of peaks) had Mann–Whitney *U*-test *p* < 0.05. After technical clustering with a threshold of 0.5, 50 features of 199 total features (25.1%) had Mann–Whitney *U*-test of *p* < 0.05. Fifteen of the 50 features with *p* < 0.05 were technical clusters which contained 35 peaks. Therefore 70 peaks (35 in 15 clusters and 35 peaks not clustered) of the original 247 peaks (28.3%) had *p* < 0.05.

### Comparison with alternative software

The vendor’s software (ProteinChip Data Manager v 3.51 was used) has a feature to identify multiply-charged peaks in individual spectra. A customizable mass error can be set as appropriate for different mass ranges. However this method does not compare the results across spectra from different samples (the algorithm is performed on each spectrum individually). In this software, a peak location (for example with an apparent m/z of 5,000) can consist of peak labels of the different samples indicating a mixture of protein labels with z = 1, 2, 3 or more (5000, 10000/2, or 15000/3). The investigator would then assign the peak label for that feature, but there would not be a built-in mechanism for clustering these assigned peaks.

The PROcess software (from the R Bioconductor suite) can be used to develop a table of biomarker peaks from SELDI spectra. The peak finding algorithm is performed on spectra obtained under uniform conditions (e.g. medium laser energy) and returns sample values for peak locations. PROcess includes an algorithm to determine whether peaks present in a vector of biomarker peaks are integer multiples of other peaks in the vector, thus potentially representing the same protein with different charges. In contrast with our Technical Clustering algorithm, there is no way to test whether peaks from different fractions, laser energies, or ProteinChip arrays could be evaluated for technical clustering.

### Implementation: fractionated samples

**Figure 4:**
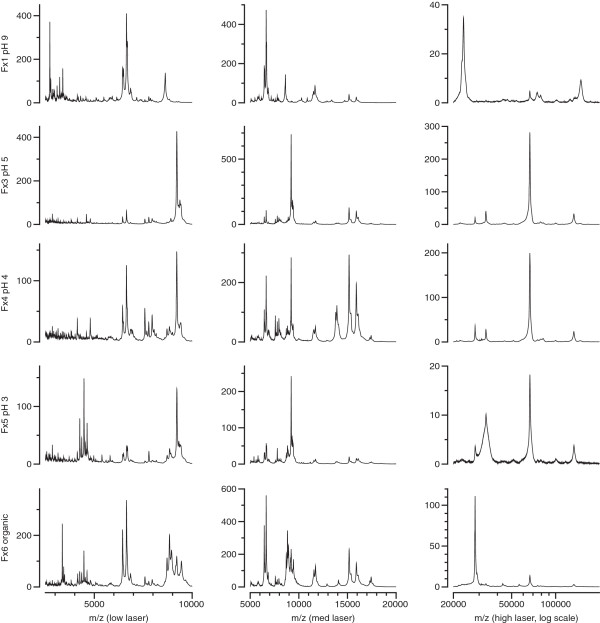
**Averaged spectra from a protein profiling biomarker disovery study.** Human plasma (85 samples, in duplicate) was chromatographed on Q anion exchange beads using a decreasing pH gradient and final organic extraction. Aliquots were applied to CM10 ProteinChip arrays and mass spectra were acquired at low (left), medium (middle), and high (right) laser energies. Each spectrum is averaged from 170 spectra.

The second demonstration dataset is from a study of fractionated plasma samples from 85 patients from 2 groups (43 and 42 patients). The spectra were obtained from five chromatographic fractions (Q anion exchange chromatography) of the samples on two types of ProteinChip arrays (CM10 and H50) from which spectra were obtained at three laser energies, essentially as described for a different dataset [14,15]. Therefore 5100 spectra were acquired in duplicate for these 85 samples under different conditions (fractions, ProteinChip arrays, laser energies). Peak intensities for 1350 peaks were obtained across the 30 conditions. To demonstrate the complexity of the dataset, some of the averaged SELDI-TOF spectra are shown in Figure 4, presented for illustration. Each panel is therefore the averaged spectrum of 170 spectra from 85 samples. The spectra are from aliquots of five chromatographic fractions of plasma applied to CM10 ProteinChip arrays, with spectra acquired using low-, medium-, and high-laser energies.

**Figure 5:**
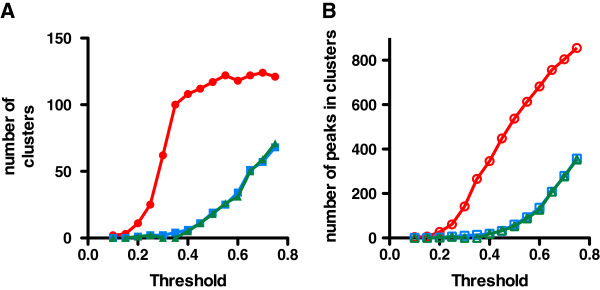
**Number of technical clusters and clustered peaks from fractionated plasma dataset as a function of algorithm variance threshold.** The Technical Clustering software was applied to a dataset of 1350 peaks from 85 fractionated samples processed in duplicate in a biomarker discovery dataset at the indicated Threshold CV. **A)** Number of technical clusters using the original table of peaks (closed red circles); peaks randomized within groups simulated data (closed blue squares); and peaks fully randomized simulated data (closed green triangles); **B)** The number of peaks within technical clusters for the original data (open red circles); simulated data using peaks randomized by sample within groups (open blue squares); and simulated data using peaks fully randomized by sample (open green triangles).

The variance threshold for technical clustering of the peaks was varied from 0.05 (5%) – 0.8 (80%) for this dataset. The number of clusters and the number of clustered peaks are shown in Figure 5. The algorithm yields clusters of peaks that have the same apparent mass on the same surface in different fractions, on multiple SELDI surfaces both within fractions and in different fractions, as well as in spectra obtained at different laser energies. The number of clusters for this dataset began to plateau at 100 clusters with a threshold of 0.35. These 100 clusters contained 346 peaks at this variance threshold.

Simulated datasets were used to test the algorithm. In the first simulated dataset, the algorithm was tested when the intensities of only one of the 1350 peaks were randomized. The intensity values for the 85 patients of the easily identified serum albumin peak of 66,680 on one surface at one laser energy in one fraction (i.e., one of 1350 peaks) were randomized, preserving the pairing of the duplicate measurements for each sample. In other words, the sample identities of a single peak’s intensities were randomized. The results of subsequent technical clustering are shown in Table 2. The m/z 66,628 peak (albumin) in Fraction 4 on CM10 ProteinChip arrays using high laser power had a contribution of 0.58 to its technical cluster with the albumin peak with z = 2 in the same fraction, array, and laser energy (m/z 33,359) at a variance threshold of 0.15 (Table 2). These two peaks formed the first technical cluster from the 1350 peaks. After randomizing the sample identity of the intensities of only the m/z 66,628 peak, neither the m/z 66,628 albumin z = 1 peak, nor the 33,359 albumin z = 2 peak clustered at that variance threshold of 0.15. The randomization of this single peak prevents it from passing the variance test of the vector of all the peaks for each patient. As can be seen in Table 2, neither the randomized 66,628 peak nor the non-randomized 33,359 peak clustered at thresholds of 0.15, 0.2 or 0.25, but finally did cluster at a CV threshold of 0.3. However, at this threshold the contribution of the randomized 66,628 peak to its technical cluster was only 0.056, a minor weighted component of the cluster. In contrast, the non-randomized 33,359 peak was the second-highest weighted component of the albumin cluster. Thus disruption of the relationship of the 66,628 peak to the other peaks in each sample diminished its correlation with the other peaks in the cluster, including the 33,359 albumin z = 2 peak from the same spectrum. At the least stringent threshold tested (0.5, chosen arbitrarily), the randomized m/z 66,628 peak contributed less than 1% (0.002) to the technical cluster for albumin that consisted of 18 peaks. At this least stringent threshold of 0.5, the non-randomized m/z 66,628 peak was still a major contributor to its technical cluster of 20 peaks in the original dataset.

**Table 2:** Randomization test of technical clustering software

**Threshold**	**Original cluster**	**Weight**	**Cluster after randomizing Fx4_CM10_high_C066628**	**Weight**
0.15	**Fx4_CM10_high_C066628_**	**0.581**	**Not clustered**	**NA**
	Fx4_CM10_high_C033359_	0.419		
0.20	Fx4_CM10_high_C0133296	0.288	**Not clustered**	**NA**
	**Fx4_CM10_high_C066628_**	**0.260**		
	Fx4_CM10_high_C033359_	0.176		
	Fx3_CM10_high_C033347_	0.129		
	Fx4_CM10_high_C022248_	0.061		
	Fx3_CM10_high_C066617_	0.053		
	Fx3_CM10_high_C022244_	0.033		
0.25	Fx4_CM10_high_C0133296	0.250	**Not clustered**	**NA**
	**Fx4_CM10_high_C066628_**	**0.230**		
	Fx4_CM10_high_C033359_	0.154		
	Fx3_CM10_high_C033347_	0.121		
	Fx3_CM10_high_C0133108	0.089		
	Fx4_CM10_high_C022248_	0.055		
	Fx3_CM10_high_C066617_	0.051		
	Fx3_CM10_high_C022244_	0.027		
	Fx4_H50_high_C022244_	0.024		
0.3	Fx4_CM10_high_C0133296	0.238	Fx3_CM10_high_C033347_	0.237
	**Fx4_CM10_high_C066628_**	**0.224**	Fx4_CM10_high_C033359_	0.237
	Fx4_CM10_high_C033359_	0.151	Fx3_CM10_high_C0133108	0.152
	Fx3_CM10_high_C033347_	0.119	Fx4_CM10_high_C022248_	0.106
	Fx3_CM10_high_C0133108	0.087	Fx3_CM10_high_C066617_	0.093
	Fx4_CM10_high_C022248_	0.054	Fx5_H50_high_C022236_	0.065
	Fx3_CM10_high_C066617_	0.050	**Fx4_CM10_high_C066628_**	**0.056**
	Fx5_H50_high_C022236_	0.030	Fx3_CM10_high_C022244_	0.028
	Fx3_CM10_high_C022244_	0.026	Fx4_H50_high_C022244_	0.026
	Fx4_H50_high_C022244_	0.020		
0.4	Fx4_CM10_high_C0133296	0.238	Fx4_CM10_high_C0133296	0.240
	**Fx4_CM10_high_C066628_**	**0.224**	Fx4_CM10_high_C033359_	0.165
	Fx4_CM10_high_C033359_	0.151	Fx3_CM10_high_C033347_	0.154
	Fx3_CM10_high_C033347_	0.119	Fx3_CM10_high_C0197893	0.117
	Fx3_CM10_high_C0133108	0.087	Fx3_CM10_high_C0133108	0.112
	Fx4_CM10_high_C022248_	0.054	Fx3_CM10_high_C066617_	0.065
	Fx3_CM10_high_C066617_	0.050	Fx4_CM10_high_C022248_	0.064
	Fx5_H50_high_C022236_	0.030	Fx5_H50_high_C022236_	0.036
	Fx3_CM10_high_C022244_	0.026	Fx3_CM10_high_C022244_	0.024
	Fx4_H50_high_C022244_	0.020	Fx4_H50_high_C022244_	0.012
			**Fx4_CM10_high_C066628_**	**0.012**
0.5	Fx4_CM10_high_C0133296	0.161	Fx4_CM10_high_C0133296	0.194
	**Fx4_CM10_high_C066628_**	**0.140**	Fx4_CM10_high_C0196854	0.155
	Fx4_CM10_high_C0196854	0.129	Fx4_CM10_high_C033359_	0.101
	Fx4_CM10_high_C033359_	0.088	Fx3_CM10_high_C033347_	0.074
	Fx3_CM10_high_C033347_	0.064	Fx3_CM10_high_C0197893	0.065
	Fx6_CM10_high_C033435_	0.060	Fx6_CM10_high_C033435_	0.065
	Fx3_CM10_high_C0197893	0.055	Fx3_CM10_high_C0133108	0.061
	Fx3_CM10_high_C0133108	0.052	Fx6_CM10_high_C066702_	0.042
	Fx6_CM10_high_C066702_	0.036	Fx6_CM10_high_C0133574	0.039
	Fx6_CM10_high_C0133574	0.033	Fx3_H50_high_C033429_	0.038
	Fx3_H50_high_C033429_	0.032	Fx4_CM10_high_C022248_	0.034
	Fx4_CM10_high_C022248_	0.030	Fx3_CM10_high_C066617_	0.033
	Fx3_CM10_high_C066617_	0.028	Fx3_H50_high_C066672_	0.023
	Fx3_H50_high_C066672_	0.019	Fx5_H50_high_C022236_	0.020
	Fx5_H50_high_C022236_	0.018	Fx3_H50_high_C0133496	0.019
	Fx3_CM10_high_C022244_	0.016	Fx3_CM10_high_C022244_	0.018
	Fx3_H50_high_C0133496	0.016	Fx4_H50_high_C022244_	0.017
	Fx4_H50_high_C022244_	0.013	**Fx4_CM10_high_C066628_**	**0.002**
	Fx5_CM10_high_C0133649	0.010		
	Fx5_CM10_high_C066776_	0.001		

The Technical Clustering software was applied to the dataset of spectra obtained from 85 samples before (left-hand column) and after (right hand column) randomizing the sample identities of the intensities of one peak (Fx4_CM10_high_C066628, in bold type). The clusters that contain the correct sample values are shown in the “Original Cluster” column at the indicated threshold CV, and after randomizing the sample intensities of just one peak on the right. The contributions (weights) of the components of the clusters are also shown.

A common use of this type of biomarker dataset is the downstream analysis for significant differences between two groups. The second and third simulated datasets were created by randomizing the sample identities for each of the peaks of the original dataset, preserving the replicate pairing of values. Thus the sample identities for the values of each peak were randomized, and the process repeated for each of the 1530 peaks. The aim was to use these randomized datasets to characterize the effect of technical clustering on downstream significance analysis. The randomization to create the second simulated dataset was performed for each peak within groups (preserving the pairing of duplicate measurements), while the randomization to create the third simulated dataset was performed for each peak without regard to groups (while still preserving the pairing of duplicate measurements). The effect of this within-peak randomizing of sample identities of values on subsequent technical clustering for these two synthetic datasets is shown in Figure 5, in comparison to the original dataset. In Figure 5, randomization of the sample identities within each peak greatly reduces the number of clusters found by the algorithm as well as the number of peaks within clusters, for both the within groups randomized data and the fully randomized data. For example, at a threshold of 0.5, the algorithm clustered 537 peaks in 117 clusters in the original dataset of 1530 peaks. In the simulated datasets, at a threshold of 0.5 the algorithm clustered 60 and 53 peaks (Figure 5B) into 19 and 18 clusters (Figure 5A) for the randomized within groups and fully randomized data, respectively.

**Figure 6:**
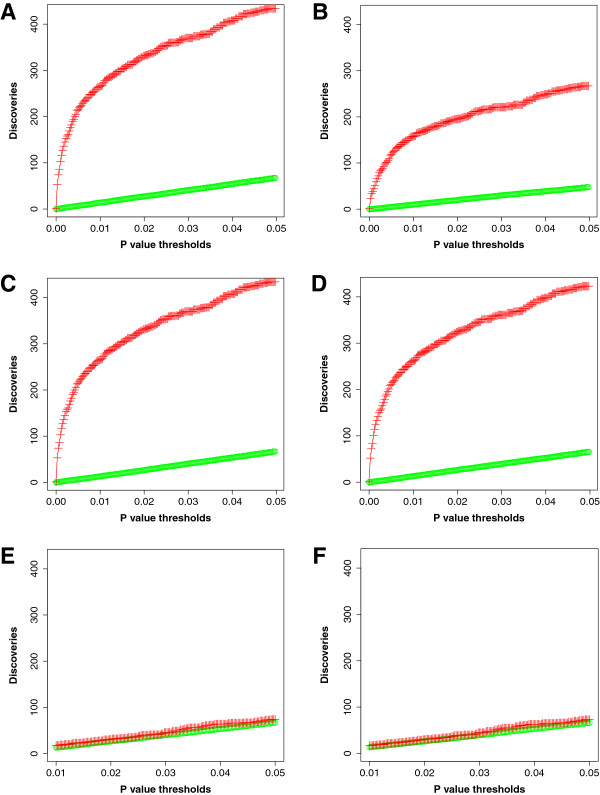
**Discoveries with Global FDR correction for Mann–Whitney *U *test.** Mann–Whitney *U* tests with FDR correction were performed on unclustered **(A**, **C**, **E)** and clustered **(B**, **D**, **F)** datasets. In each panel, the number of discoveries with *p* < 0.05 is plotted in red crosses, while the average number of discoveries of the data permuted 100 times is plotted with open green circles. **A)** original dataset of 1350 peaks; **B)** original dataset after technical clustering at threshold 0.5; **C)** simulated dataset of each peak randomized within groups; **D)** simulated dataset from **C** after technical clustering at threshold 0.5; **E)** simulated dataset of each peak fully randomized; **F)** simulated dataset from **E** after technical clustering at threshold 0.5.

To characterize the effects of clustering on subsequent analysis of significant differences between groups, we performed the Mann–Whitney *U* test on the original data and the data after Technical Clustering [13]. Before technical clustering, 434 of 1350 peaks (32%) in the original dataset had a Mann–Whitney *U* test *p* < 0.05 (red line for Discoveries in Figure 6A). After technical clustering at a threshold of 0.5, 267 of 930 features (29%) had *p* < 0.05 (Figure 6B). These 267 features consisted of 38 technical clusters with *p* < 0.05 containing 176 peaks, with the remaining significantly different features being 229 peaks not clustered at this threshold. Therefore, after technical clustering, 405 of the original 1350 peaks were p < 0.05 (combining peaks within clusters and peaks not clustered), compared to 434 peaks when the data weren’t clustered. Therefore, essentially equal proportions of original peaks were significantly different when comparing unclustered and clustered data.

For the simulated dataset in which sample identities are randomized within peaks within groups, the Mann–Whitney *U* test results were identical to the original dataset, as expected (Figure 6C). After technical clustering at a threshold of 0.5, this within group randomized dataset had 423 of 1309 features with *p* < 0.05. There were only 3 technical clusters among the 423 features with *p* < 0.05, composed of 8 peaks (Figure 6D). This result demonstrates that the algorithm is sensitive to disruption of the values vector for each sample. Overall, the two groups remain highly significantly different because the randomization was performed only within groups. However, at this threshold, the algorithm clustered very few peaks (Figure 5). In contrast, fully randomizing the sample identities for each peak of the original dataset yields randomized groups of sample values. Therefore no significantly different peaks were found after false discovery rate correction (the green symbols in Figure 6) either before (Figure 6E) or after technical clustering at a threshold of 0.5 (Figure 6F).

### Utility of clustering technical aliases

Proteomic studies such as biomarker discovery often contain many more features than samples, and contain features that correlate either biologically or are technical aliases. Our previous clustering algorithm did not distinguish between biological and technical aliases [7], while this present algorithm is a tool for correlating technical aliases only. The correlation algorithms are similar, with the difference being that this current effort tests whether peak masses are multiples of the mass of other peaks (as found in the same protein having different charges, or protein aggregation) or are the same as peaks of similar apparent mass in spectra obtained at different laser energies, in chromatographic fractions and/or on multiple surfaces.

**Figure 7:**
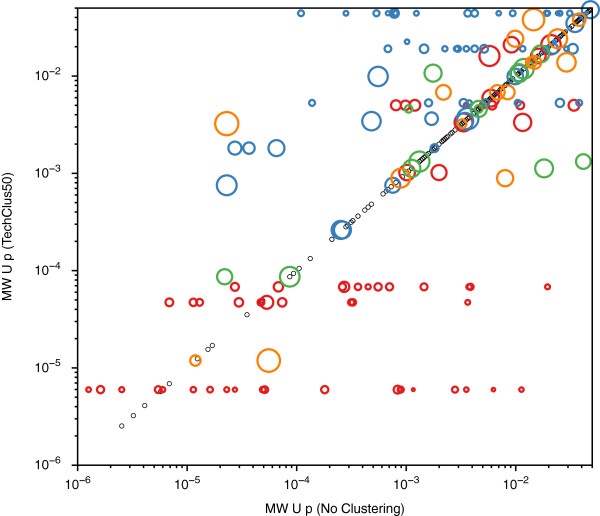
**Comparison of Mann–Whitney *U *test results before and after technical clustering.** The Mann–Whitney *U* test results for the original data are plotted on the abscissa, and the *U* test results after technical clustering at threshold 0.5 are plotted on the ordinate. For convenience of viewing, two peaks with *p* < 1 × 10^-7^ and 972 peaks with *p* > 0.05 are not plotted. Peaks with *p* < 0.05 that do not cluster at variance threshold 0.5 are represented as small black open circles, and fall on the diagonal. The clustered peaks with *p* < 0.05 are represented as a bubble plot, with the area of the bubble being proportional to the weight (contribution) of that protein peak to that cluster’s centroid. For viewing convenience, clusters have bubbles of different colors.

One utility for using the technical clustering algorithm in a biomarker discovery study can be seen in Figure 7. In this bubble chart, the Mann–Whitney *U* test results with *p* < 0.05 for technically clustered features (threshold = 0.5) are plotted versus the corresponding Mann–Whitney U test results for the original feature set. The *p* value of an individual cluster therefore appears as horizontal lines of bubbles that correspond to the original *p* values of the members of that cluster. The area of the bubbles is proportional to the weight of that protein peak to that cluster. Peaks that did not cluster at this threshold fall on the diagonal line at their respective unchanged *p* value. For example, a prominent cluster that is highly significant with a *p* = 6 × 10^-6^ can be seen to consist of 20 peaks with original *p* values ranging from 0.01 to 0.000001. The highest weighted peak in the cluster has an apparent mass of 17,419 on H50 surfaces in fraction 5 (pH 3 extraction of anion exchange chromatography of the original samples) at medium laser energy. Most of the alias peaks in this cluster have apparent mass of approximately 8,710 (z = 2 for 17,419). Evaluation of the results before and after technical clustering allows the investigator to rationally choose which peaks represent the relevant species for further identification, rather than using more subjective judgement. Biological identification and interpretation of the biology of the biomarker can be focused solely on the 17,419 peak, thereby reducing the complexity of that phase of the discovery study.

**Figure 8:**
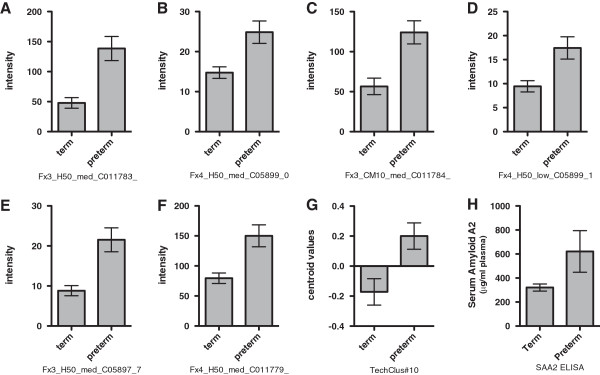
**Test results from one technical cluster from a biomarker discovery dataset of a mouse preterm labor model.** The intensities of six SELDI-TOF MS peaks for term labor and preterm labor are shown in **A** – **F**. These peaks formed a technical cluster at a variance threshold of 0.3. The cluster centroids for term and preterm labor for this cluster is shown in **G**. The subsequent ELISA assay results for serum amyloid A2 are shown in **H**, after identification of candidate biomarker by chromatographic purification, gel purification, and sequencing of tryptic fragments as previously described in the text. Panels **A** - **G** are mean ± sem with n = 7 for Term and n = 6 for Preterm. Panel **H** is mean ± sem with n = 5 for both Term and Preterm.

In one of our previous reports about a search for markers of premature birth in a mouse model, we found biomarkers in plasma using SELDI-TOF-MS profiling of fractionated plasma [15]. In these studies several peaks of approximately 11,700, 11,800, 5,800, and 5,900 were significantly different when comparing the plasma of control mice and mice that gave birth prematurely after injection with lipopolysaccharide (LPS). A subsequent experiment compared the plasma of mice injected with a lower concentration of LPS, resulting in half of the mice giving birth at term, and half giving birth preterm. The same peaks were higher in the preterm group than in the term group. Thus, higher amounts of this biomarker correlated with preterm vs term birth in this model. After using this algorithm, technical clusters consisted of the 11,800 and 5,900 peaks, and of the 11,700 and 5,800 peaks (Figure 8). This result provided a rationale for focusing on purification of the 11,700 and 11,800 peaks. After trypsinization and MS/MS analysis the peaks were identified as serum amyloid A1 and serum amyloid A2. Subsequent analysis of the plasma serum amyloid A2 content in the plasma confirmed that the levels were significantly higher in the preterm group, in both the control vs preterm study, and the subsequent correlation study between term and preterm birth following low dose LPS (Figure 8). This software gave us a rational, rather than strictly subjective basis as to which biomarker peaks should be identified, and which were technical aliases of the same protein.

In another of our previous reports, we identified several proteins in rat CSF that were presymptomatic biomarkers for primary glioma [12]. Seven of the top 11 significantly different peaks in that study were technical aliases of three of the other four peaks after using this algorithm. Subsequent biochemical identification of the proteins with z = 1 confirmed the identity of the technical aliases, and subsequent orthogonal assays confirmed the statistically difference between the groups.

The observation that a peak is never found in any technical cluster can also provide useful information in a discovery study. In our published work on CSF from rats in a primary glioma model, one of the peaks (m/z = 3493) of highest significance did not cluster even at a high variance threshold of 0.8 using the technical clustering algorithm. This confirmed that identification strategies for this biomarker could be confidently focused on this low molecular weight protein, which we eventually biochemically identified as a novel fragment of α1-macroglobulin [12].

There are limitations to the use of this algorithm. There is no correct variance threshold for technical clustering, nor is perfect technical clustering likely achievable. This algorithm has been developed for biomarker discovery studies using protein profiling datasets obtained with the SELDI-TOF-MS platform. Among the advantages of this platform are its sensitivity and capability of reasonably high throughput of samples. Among the disadvantages of this platform are the modest mass resolution and mass accuracy. In addition, identification of all potential discoveries from this platform requires additional biochemical, proteomic, and immunological techniques, since identification cannot be achieved directly from the mass spectra. Nonetheless, this present algorithm is a useful tool for decreasing the subjectivity of one aspect of the analysis of these large datasets.

## Conclusions

The approach presented here describes a framework to rationally correlate the technical aliases present in SELDI proteomic biomarker discovery datasets. Representing each cluster through only its centroid helps reduce the complexity of the dataset and reduces the number of statistical tests applied to the dataset. Using clustering will also help highlight relationships between technically-correlated peaks. This algorithm is a useful tool as part of multi-faceted analyses of biomarker discovery datasets, such as in protein profiling SELDI-TOF-MS studies.

## Methods

SELDI peak intensity datasets obtained on rat, mouse, and human samples were used to develop and demonstrate the algorithm described in this study. The rat CSF [12] and mouse plasma [15] samples were obtained in studies approved by the Stanford University IUCAC in accordance with guidelines for animal safety and welfare. The dataset developed from human plasma samples was obtained in studies approved by the Stanford University Institutional Review Board (protocol IRB-13965). Informed consent was obtained from the parents of all subjects and assent from all subjects > 6 years of age.

All mass spectra were obtained using a Bio-Rad PCS4000 SELDI mass spectrometer. Aliquots of CSF (4 μl) were denatured with urea and applied to CM10 ProteinChip arrays (weak cation exchange surface) as described in [12]. Mass Spectra were acquired using low (3200 nJ), medium (4200 nJ), and high (7200 nJ) laser energy. The mass spectra were externally calibrated using Bio-Rad’s protein standards. Spectra were pre-processed using noise reduction and baseline subtraction in ProteinChip Data Manager 3.51. The signal of spectra acquired using the same conditions (laser energy) were normalized. Peaks were detected using ProteinChip Data Manager, and the resulting peak tables exported as text files without averaging replicate values of samples. The output files were then combined to create the dataset used for technical clustering.

Aliquots of plasma (20 μl) were denatured with urea, applied to strong anion-exhange Q ceramic HyperD F beads (Pall) as described in [15]. The beads were extracted with 200 μl buffer in a decreasing pH gradient, followed by a final acidic organic extraction. A small amount of each fraction (10 μl) was bound to CM10 weak cation exchange or H50 reverse phase ProteinChip arrays as described [15]. Mass spectra were acquired and processed as above.

The averaged spectra depicted in the figures were created using our Simultaneous Spectrum Analysis software [11]. Mass calibrated spectra (without other processing) were exported as text files. Spectra acquired under the same conditions (laser energy, fraction and ProteinChip array) were processed in the software to create an average spectrum for that condition. Figures of the averaged spectra were created using Datagraph 3.1.1 software (Visual Data Tools).

### Availability and requirement

**Project Name:** Technical Clustering

**Project home page:**http://med.stanford.edu/labs/harvey_cohen/

**Operating system(s):** Platform independent

**Programming language:** R (tested in version 2.15.1)

**Other Requirements:** R packages “gsubfn”, “proto”, and “pamr” and their dependencies must be installed.

## Abbreviations

SELDI-TOF-MS: Surface enhanced laser desorption ionization – time of flight mass spectrometry.

## Competing interests

The authors have no financial or non-financial competing interests.

## Authors’ contributions

JW and HC directed the research; SR wrote the R code; JW, SR, and HC wrote the paper. All authors read and approved the final manuscript.
